# Adeno-Associated Virus-Mediated Single-Cell Labeling of Mitral Cells in the Mouse Olfactory Bulb: Insights into the Developmental Dynamics of Dendrite Remodeling

**DOI:** 10.3389/fncel.2020.572256

**Published:** 2020-12-09

**Authors:** Kazuya Togashi, Masato Tsuji, Shunsuke Takeuchi, Ryota Nakahama, Hiroyuki Koizumi, Kazuo Emoto

**Affiliations:** ^1^Department of Biological Sciences, Graduate School of Science, The University of Tokyo, Tokyo, Japan; ^2^International Research Center for Neurointelligence (WPI-IRCN), The University of Tokyo, Tokyo, Japan

**Keywords:** olfactory sensory system, mitral cells, neuronal remodeling, adeno-associated virus, dendrite, pruning

## Abstract

Neurons typically remodel axons/dendrites for functional refinement of neural circuits in the developing brain. Mitral cells in the mammalian olfactory system remodel their dendritic arbors in the perinatal development, but the underlying molecular and cellular mechanisms remain elusive in part due to a lack of convenient methods to label mitral cells with single-cell resolution. Here we report a novel method for single-cell labeling of mouse mitral cells using adeno-associated virus (AAV)-mediated gene delivery. We first demonstrated that AAV injection into the olfactory ventricle of embryonic day 14.5 (E14.5) mice preferentially labels mitral cells in the olfactory bulb (OB). Birthdate labeling indicated that AAV can transduce mitral cells independently of their birthdates. Furthermore, in combination with the Cre-mediated gene expression system, AAV injection allows visualization of mitral cells at single-cell resolution. Using this AAV-mediated single-cell labeling method, we investigated dendrite development of mitral cells and found that ~50% of mitral cells exhibited mature apical dendrites with a single thick and tufted branch before birth, suggesting that a certain population of mitral cells completes dendrite remodeling during embryonic stages. We also found an atypical subtype of mitral cells that have multiple dendritic shafts innervating the same glomeruli. Our data thus demonstrate that the AAV-mediated labeling method that we reported here provides an efficient way to visualize mitral cells with single-cell resolution and could be utilized to study dynamic aspects as well as functions of mitral cells in the olfactory circuits.

## Introduction

Mammalian olfactory sensory neurons relay odor information to the olfactory bulb (OB), where olfactory sensory axons form synapses with dendrites of mitral cells and tufted cells, the second-order projection neurons in the OB (Malun and Brunjes, 1996; Blanchart et al., 2006). In turn, mitral cells and tufted cells receive and convey the odor information to higher cortical regions. In the adult OB, mitral cells extend radially a single apical dendrite that arborizes a tuft within one glomerulus (Mori and Sakano, 2011; Murthy, 2011; Sakano, 2020). Also, mitral cells extend lateral dendrites that are widely distributed within a horizontal plane in the external plexiform layer and make reciprocal dendrodendritic synapses with granule cells. During perinatal development, however, mitral cell dendrites undergo extensive remodeling: mitral cells initially extend multiple dendritic branches to multiple glomeruli and subsequently lose all but one dendritic branch to maintain contact with a single glomerulus as they mature (Lin et al., 2000; Inoue et al., 2018). This dendrite remodeling is thought to require neural activity (Wong and Ghosh, 2002) and Notch activity (Muroyama et al., 2016) in mitral cells, but mechanisms underlying the dendrite remodeling in mitral cells are still incompletely understood.

One reason for our limited knowledge is the lack of a convenient method to visualize and manipulate mitral cells *in vivo*. Conventional methods used to visualize mitral dendrite morphology of rodent mitral cells rely on stochastic labeling by retrograde labeling using lipophilic dyes *via* the lateral olfactory tract (LOT; Malun and Brunjes, 1996; Lin et al., 2000; López-Mascaraque et al., 2005; Blanchart et al., 2006). Also, *in utero* electroporation has been recently utilized to induce ectopic gene expression in developing mitral cells (Imamura and Greer, 2015; Muroyama et al., 2016). *In utero* electroporation typically introduces plasmids into mitotically active mitral/tufted cell precursors, which are surrounding the embryonic ventricle in the OB (Imamura and Greer, 2013). Therefore, *in utero* electroporation is often applied to label subpopulations generated in a homogeneous time window (Imamura and Greer, 2015). Also, a previous report showed that the distributions of the early-born and the late-born mitral cells are partially segregated within the OB, suggesting that the localization of mitral cells in the OB is also biased with the timing of neurogenesis (Imamura et al., 2011; Imamura and Greer, 2015). It is thus likely that *in utero* electroporation tends to label a limited population of mitral cells with homogenous birthdates and localization within the OB.

A convenient method for birthdate-independent labeling of mitral cells should be helpful for global analysis of the mitral population as well as for functional manipulation of mitral cells. One candidate approach to this labeling involves the adeno-associated virus (AAV), which provides an efficient approach to gene delivery in the nervous system (Haery et al., 2019). AAV is a naturally replication-defective, nonpathogenic, single-stranded DNA virus (Kaplitt et al., 1994). The single-stranded DNA of the AAV genome consists of two open reading frames, *rep* and *cap*, and the inverted terminal repeats (ITRs) at both ends of the DNA strand. The ITRs are cis-acting elements necessary for virus replication, packaging, and integration (Musatov et al., 2002). Recombinant AAV vectors can be generated by co-transfecting host cells with a plasmid containing a transgene expression cassette flanked by the cis-acting ITRs and a plasmid expressing the *rep* and *cap* genes in trans, in the presence of a helper virus gene (Samulski et al., 1989). Previous reports indicate that the recombinant AAV vectors permit nontoxic transduction and long-term gene expression in neurons (McCown et al., 1996; Murlidharan et al., 2014). Furthermore, an important feature of AAV-mediated gene transfer is that, unlike *in utero* electroporation, AAV vectors can efficiently transduce both post-mitotic neurons and mitotically active cells (Haery et al., 2019). Therefore, AAV vectors should be suitable for the transduction of mitral cells at any stage in the cell cycle, independently of birthdates. To date, however, AAV-mediated gene transfer methods have not yet been applied to mitral cell labeling in the developing mammalian OB.

In this study, we aimed to develop an AAV-mediated method of labeling mitral cells with single-cell resolution. We demonstrated that injecting AAV vectors into the olfactory ventricle of an embryonic day 14.5 (E14.5) mouse yields a preferential expression of the reporter EGFP in developing mitral cells. Further analyses indicated that AAV injection at E14.5 can label mitral cells generated in E9–13 stages, suggesting that the AAV-mediated gene transfer transduces mitral cells independently of their birthdates. Using this AAV-mediated labeling method, we found that ~50% of mitral cells complete dendrite remodeling before birth. Also, we found an atypical subtype of mitral cells that have multiple dendritic shafts innervating the same glomeruli. Thus, the AAV-mediated labeling method that we reported here provides an efficient way to visualize mitral cells in a single cell resolution and could be utilized to study dynamic aspects as well as functions of mitral cells in the olfactory circuits.

## Materials and Methods

### Animals

All animal experiments were carried out following the regulations and guidelines for the care and use of experimental animals at the University of Tokyo and were approved by the University of Tokyo Graduate School of Science. Pregnant ICR mice were purchased from Japan SLC Inc.

### Cell Culture

AAV-293 cells (Agilent Technologies) were grown in Dulbecco’s modified Eagle’s medium (DMEM; Sigma–Aldrich, D5796) supplemented with 10% of fetal bovine serum (GE Healthcare/HyClone), 50 units/ml of penicillin, and 50 mg/ml of streptomycin (Thermo Fisher Scientific), and 2 mM GlutaMax I (Thermo Fisher Scientific) at 37°C in a 5% CO_2_ humidified incubator according to the manufacturer’s instructions.

### Plasmid Construction

Plasmids to produce AAV were as follows: the AAV Helper-Free system (included pHelper and pAAV-RC) was purchased from Agilent Technologies; pAAV-DJ was from Cell Biolabs. The following plasmids were obtained from Addgene: pAAV-CAG-GFP was a gift from Edward Boyden (Addgene # 37825^1^; RRID:Addgene_37825) tTA IRES GFP was a gift from Scott Lowe (Addgene # 18783^2^; RRID:Addgene_18783); paavCAG-iCre was a gift from Jinhyun Kim (Addgene plasmid # 51904^3^; RRID:Addgene_51904; Druckmann et al., 2014); pscAAV-GFP was a gift from John T Gray (Addgene plasmid # 32396^4^ RRID:Addgene_32396; Gray and Zolotukhin, 2011). pAAV-CAG-tTA was generated by amplifying the full-length coding region of tTA cDNA and inserting it into pAAV-CAG-GFP in place of EGFP. pscAAV-TRE-iCre-myc plasmid construction as follows: The CMV promoter region was replaced with the tetracycline response element (TRE) amplified from pTRE (Clontech) by PCR. Subsequently, the DNA fragment encoding GFP was replaced with Myc-tagged iCre amplified from paavCAG-iCre by PCR. Finally, the woodchuck hepatitis virus posttranscriptional regulatory element (WPRE) excised from pAAV-CAG-GFP was inserted between the full-length coding sequence of iCre-myc and SV40 polyA at NotI.

### Transfection and AAV Preparation

Transfection was performed using a calcium phosphate co-precipitation method based on previous reports (Dudek et al., 1998; Okada, 2013) with slight modifications. In brief, 2 days before transfection, 3 × 10^6^ each of AAV-293 cells was reseeded in four T-150 flasks and cultured to 60–70% confluency in 18 ml of culture medium. One to four hours before transfection, a half volume of the culture medium was replaced with a fresh growth medium. Subsequently, 60 μg each of the plasmid (expression vector, *Rep/Cap* vector, and pHelper) was mixed well with 6 ml of 0.3 M CaCl_2_ solution and then finally combined with an equal amount of 2 × HBS. Three ml each of the final solution was applied to each culture flask. Following a 4-h incubation in a 5% CO_2_ incubator, the supernatant was replaced with DMEM/F-12 Ham’s medium (Sigma–Aldrich) containing 2% FBS, 50 mg/ml of streptomycin, and 2mM GlutaMAX I (Thermo Fisher Scientific) and incubated at 37°C in a 10% CO_2_ humidified incubator. Cells and culture medium were harvested 2–3 days after transfection, and AAV was purified using the AAV Purification ViraKit (ViraPur LLC) following the manufacturer’s manual. The details of the AAV preparation protocol are available upon request. The virus concentration was quantified using AAVpro^®^ Titration Kit (Takara Bio) and used at a concentration of ~1.5 × 10^13^ vg/ml.

### AAV Injection

AAV injection was performed essentially as in utero electroporation described previously (Koizumi et al., 2017). In brief, AAVs were injected into the embryonic olfactory ventricle at E14.5 because the mouse OB becomes visible from E14 when viewed from the parietal side. We performed the injection using a glass microcapillary with a tip diameter of 30 μm that was sharpened to 20 degrees with a micro grinder. Each capillary was inserted perpendicularly from the parietal region of the embryo to the center of the OB. The depth and injection volume were controlled empirically. The injection volume at once was determined empirically and estimated to be less than 0.1 μl by measuring the residual virus solution after injection. Fibroblast-like cells and endothelial cells outside the brain were occasionally labeled presumably due to leakage of the AAV solution from the ventricle. These non-neuronal cells were easily distinguished from neurons by the morphology and the location of cells. We performed the AAV-mediated mitral cell labeling using AAV-DJ serotype, except for Figure 2 in which we examined multiple serotypes of AAVs including AAV2, AAV-DJ, AAV-DJ/8, and AAV-rh10 (kindly provided by Drs. Kuroda and Kaibuchi at Nagoya University).

**Figure 1:**
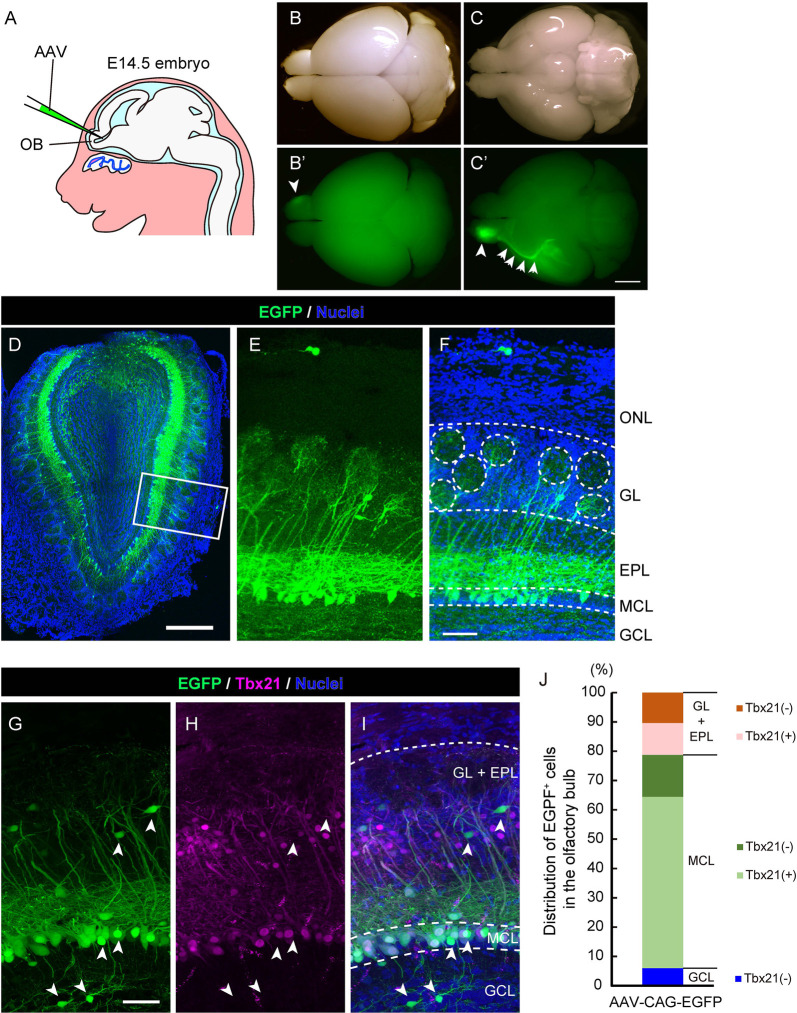
Adeno-associated virus (AAV) injection into the olfactory ventricle preferentially labels mitral cells in the mouse olfactory bulb (OB). **(A)** A schematic view illustrating the way for AAV injection into the mouse embryonic olfactory ventricle using a glass microcapillary. **(B,C)** Photomicrographs show top **(B)** and bottom **(C)** views of an AAV-injected brain from a mouse at P14. The corresponding fluorescent images are shown in **(B′)** and **(C′)**. Scale bar, 2 mm. EGFP signal is observed not only in the right OB (arrowhead) but also in the axon bundle called the lateral olfactory tract (LOT; arrows). **(D–F)** A representative image of a coronal OB section from an AAV-injected mouse at 3 weeks of age. Scale bar, 500 μm. The magnified image of the boxed area is shown in **(E)** and **(F)**. Scale bar, 100 μm. The typical layer structure was visualized by nuclear staining shown in blue. ONL, olfactory nerve layer; GL, glomerular layer; EPL, external plexiform layer; MCL, mitral cell layer and GCL, granule cell layer. **(G–I)** The coronal section was immunostained with anti-GFP (green) and anti-Tbx21 (magenta), a marker for mitral cells in the OB, respectively. Scale bar, 50 μm. EGFP-positive but Tbr21-negative cells are indicated by arrowheads. Based on nuclei and Tbx21 staining **(G–I)**, layers in the OB were divided into three parts; GL + EPL, MCL, and GCL. **(J)** Distribution of EGFP-positive cells in the OB. The numbers of EGFP-positive cells and EGFP-positive cells expressing Tbx21 were counted, and percentages of Tbx21-positive and Tbx21-negative/EGFP-positive cells among total EGFP-positive cells were calculated in each part. We counted 301 EGFP-positive cells from five mice (three males and two females).

**Figure 2:**
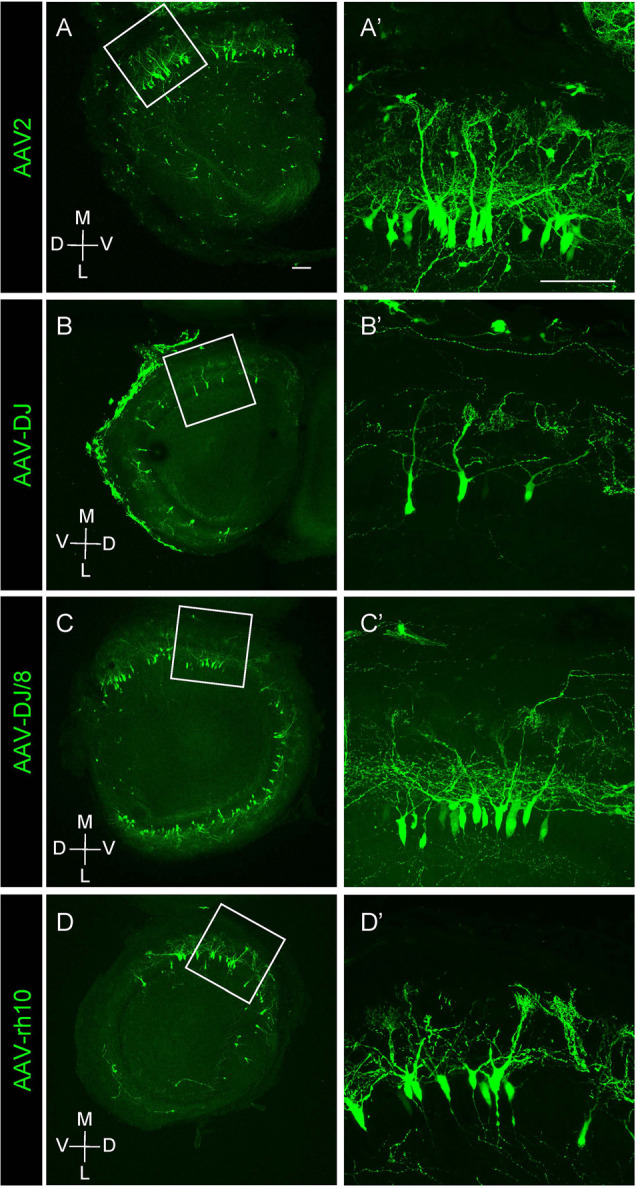
Expression of different AAV serotypes in mouse mitral cells. **(A–D)** Gene transduction to mitral cells by AAV2, AAV-DJ, AAV-DJ/8, and AAV-rh10. The coronal sections of the OB from P1 pups transduced by each AAV serotype showed no obvious difference for the neuronal tropism. The dorsal-ventral axis and the medial-lateral axis are indicated. Panels **(A′–D′)** are magnified images of the corresponding boxed area in the left panels **(A–D)**. Scale bar, 100 μm.

### Antibodies and Fluorescent Dyes

Antibodies used in this study are as follows: Goat anti-GFP antibody (Genetex, GTX26673, 1:1,000 or Abcam, ab5450, 1:1,000); rabbit anti-Tbx21 antibody [a gift from Dr. Yoshihara (Mizuguchi et al., 2012), 1:10,000]; Alexa488-conjugated donkey anti-goat IgG (Thermo Fisher Scientific, A-11055, 1:1,000) and Alexa 555-conjugated donkey anti-rabbit IgG (Thermo Fisher Scientific, A-31572, 1:1,000) or Alexa 647-conjugated donkey anti-rabbit IgG (Thermo Fisher Scientific, A-31573, 1:1,000) were used as secondary antibodies for detection of immunofluorescence. Nuclei were counterstained by DAPI (Roche, Figures 1D,F) or DRAQ5 (BioStatus, Figures 1I,2) following the manufacturer’s instructions.

### Birthdate Determination by EdU

Each pregnant dam with E9 to E13 fetuses was intraperitoneally injected with EdU dissolved in PBS at a concentration of 25 mg/kg each. The P0 newborn mice were perfused transcardially with PBS containing 4% paraformaldehyde, and small cranial incisions were made to expose the brains to the fixative. Mice were subsequently submerged in fixative overnight at 4°C. Then, brains were removed and frozen, and the frozen tissue was cut into 100 μm thick sections using a cryostat. Finally, EdU signals were developed by Click-iT Plus EdU Alexa Fluor 555 Imaging Kit (ThermoFisher Scientific) according to the manual.

### Brain Slice Preparation

All the mice used in the histological studies were perfused transcardially with 4% paraformaldehyde in PBS. The brains were removed and post-fixed overnight in the fixative at 4°C. Then, the solution was replaced with PBS containing 20% sucrose and incubated for 12 h at 4°C. Next, brains were further incubated in PBS containing 30% sucrose for 12 h at 4°C, and subsequently embedded in OCT compound in cryomolds and stored at −80°C until needed. Unless otherwise noted brains were sectioned at a thickness of 100 μm on a cryostat.

### Image Analysis

The microphotographs of brain slices were taken on a Leica TCS SP8 confocal microscope (Leica Microsystems). The images were obtained at 5.0 or 0.8 μm *z*-intervals using 10× [numerical aperture (NA) 0.4] or 40× [NA 1.3] objectives, respectively. The macroscopic brain images in Figures 1B,C were taken using a fluorescent dissecting microscope MZ10F equipped with a DFC7000T CCD camera (Leica Microsystems). The 3D images were analyzed using LAS X (Leica Microsystems) and Imaris (Bitplane).

### Quantitative Analysis and Statistics

For Figure 5, EGFP-positive mitral cells were imaged by Leica TCS SP8 confocal microscope (Leica Microsystems), and the 3D image reconstruction was performed using LAS X (Leica Microsystems) and Imaris (Bitplane). Dendrite morphology was analyzed focusing on the EGFP-positive mitral cells whose soma and the apical dendrites were both fully visible within a given 100 μm slice. We classified the “mature type”, “separated type”, and the “converged type” mitral cells based on the apical dendrite morphology as following: the “mature type” cells have a single tufted apical dendrite that innervates the glomerulus layer; the “separated type” cells have multiple tufted apical dendrites that innervate multiple different places in the glomerulus layer, and the “converged type” cells have multiple tufted apical dendrites that innervate a single place in the glomerulus layer. The center of gravity of each slice from the coronal sections was determined and calculated the cellular location in each plane as the azimuth distribution using ImageJ software. For Figures 6E–I, we quantified the number of the neurites arising from the soma, including axons and dendrites, using the 3D reconstruction software LAS X (Leica Microsystems) and Imaris (Bitplane). We defined the neurite number of given neurons by checking the 3D images from at least three distinct angles (see Supplementary Figure 1). The statistical analysis was performed with the Kruskal–Wallis test followed by the Steel–Dwass *post hoc* test for multiple comparisons. All statistical analysis was performed using R software. Rao’s spacing test is a popular non-parametric statistic for testing the uniformity of circular data. The basic idea of this test is that if the underlying distribution is uniform, successive observations should be approximately evenly spaced. Large deviations from this distribution, resulting from unusually large or small spaces, are considered evidence for directionality. The test statistic *U* is essentially the sum of the deviations of the actual arc lengths from this expectation, which is defined as:

$$U=\frac{1}{2}\sum_{i=1}^{N}\left|T(i)-\lambda \right|$$

where

*λ* = 360/*N*

*T*(*i*) = *f*(*i* + 1) − *f*(*i*) for 1 ≤ *i* ≤ (*n* − 1)

and

*T*(*n*) = (360 − *f*(*n*)) + *f*(*i*) for *i* = *n*

Thus, a sufficiently high test statistic suggests directionality.

**Figure 3:**
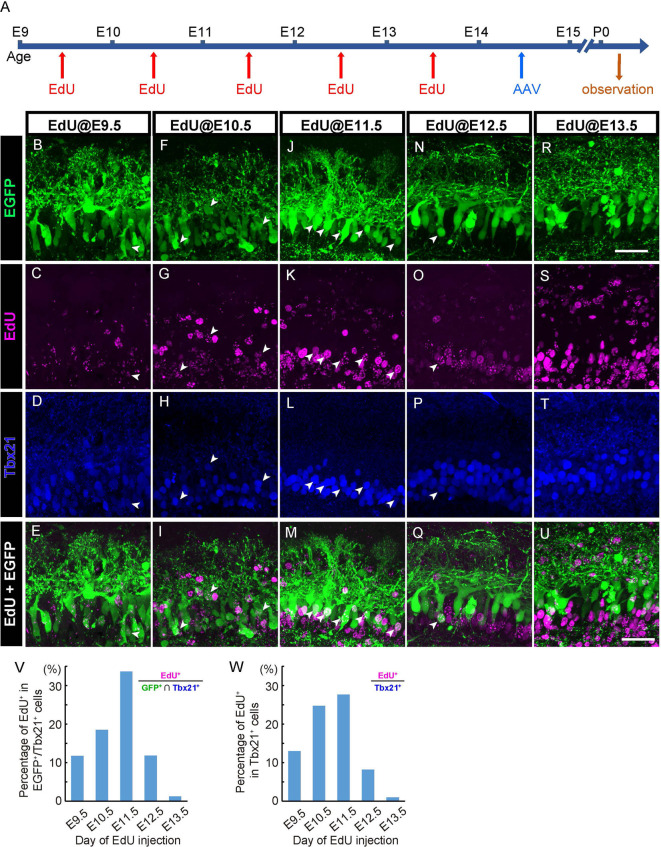
AAV transduces mitral cells independently of birthdates. **(A)** The diagram shows the time course of EdU and AAV injection, and preparation. **(B–U)** Each column corresponds to the timing of EdU injection (E9.5, E10.5, E11.5, E12.5, and E13.5, respectively). Immunohistochemistry against EGFP (**B,F,J,N**, and **R**, green) and EdU (**C,G,K,O**, and **S**, magenta) were examined with Tbx21 (**D,H,L,P**, and **T**, blue) detection at P0. EdU^+^ cells of EGFP^+^/Tbx21^+^ cells are indicated by arrowheads. Merged images (EGFP and EdU) are shown at the bottom (**E,I,M,Q**, and **U**). (**V,W**) The timing of administration of EdU and the ratio of EdU-labeled cells in EGFP-positive and Tbx 21 positive cells **(V)**. The numbers of mice analyzed are 1, 1, 3, 1, and 3 for E9.5, E10.5, E11.5, E12.5, and E13.5, respectively. The timing of administration of EdU and the ratio of EdU-labeled cells in Tbx21-positive cells **(W)**. The numbers of mice analyzed are 1, 2, 2, 2, and 2 for E9.5, E10.5, E11.5, E12.5, and E13.5, respectively. All pups examined in these studies were selected based on GFP labeling under fluorescence dissection microscopy when we fixed and extracted the brain, and thus sex was not determined. Scale bar, 100 μm.

**Figure 4:**
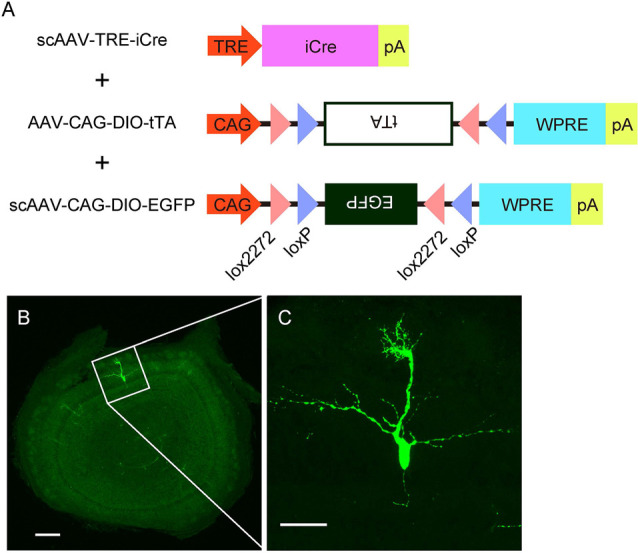
AAV-mediated single-cell labeling of mitral cells. **(A)** A schema illustrates the viral contracts used in this study. **(B)** A representative image of a coronal OB section from an AAV-injected mouse at P3. Scale bar, 200 μm. The magnified image of the boxed area is shown in **(C)**. Scale bar, 50 μm.

**Figure 5:**
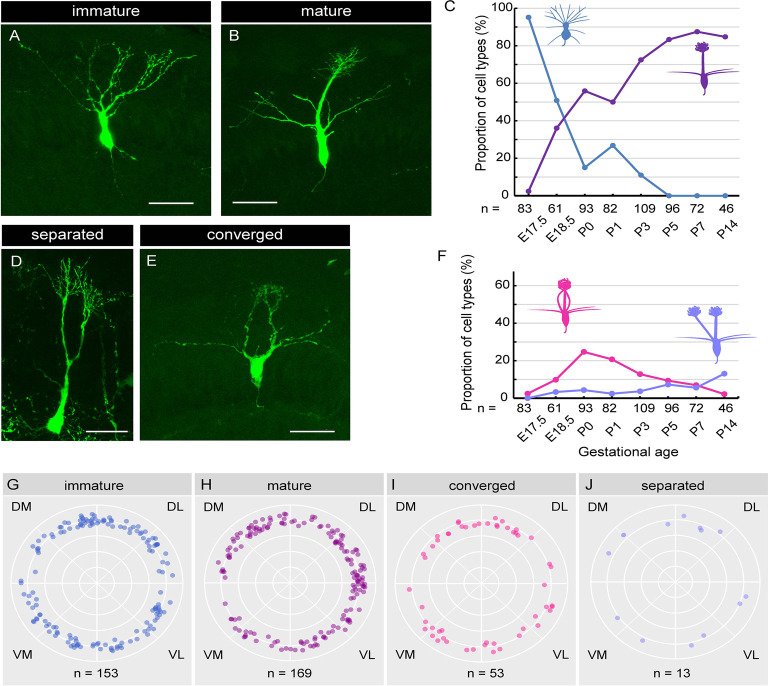
Single-cell analysis of dendrite development in mitral cells. **(A,B)** Representative images of “immature type” **(A)** and “mature type” **(B)** mitral cells, respectively. **(C)** The perinatal changes in the proportions of “immature type” and “mature type” mitral cells. **(D,E)** Representative images of “separated type” **(D)** and “converged type” **(E)** mitral cells, respectively. **(F)** The perinatal changes in the proportions of “separated type” and “converged type” mitral cells. **(G–J)** Azimuth distribution of AAV-labeled mitral cells with immature **(G)**, mature **(H)**, converged **(I)**, and separated **(J)** dendrites. Rao’s spacing test of uniformity showed no significant deflection in the coronal sections. DM, dorsomedial; DL, dorsolateral; VM, ventromedial; VL, ventrolateral. The numbers of mice counted are 10, 6, 13 (five males and eight females), 14 (seven males and seven females), 10 (four males and six females), 7 (three males and four females), 7 (four males and three females), and 5 (two males and three females) for E17. 5, E18.5, P0, P1, P3, P5, P7 and P14, respectively. The sex of pups analyzed at E17.5 and E18.5 was not determined. Scale bars, 50 μm.

**Figure 6:**
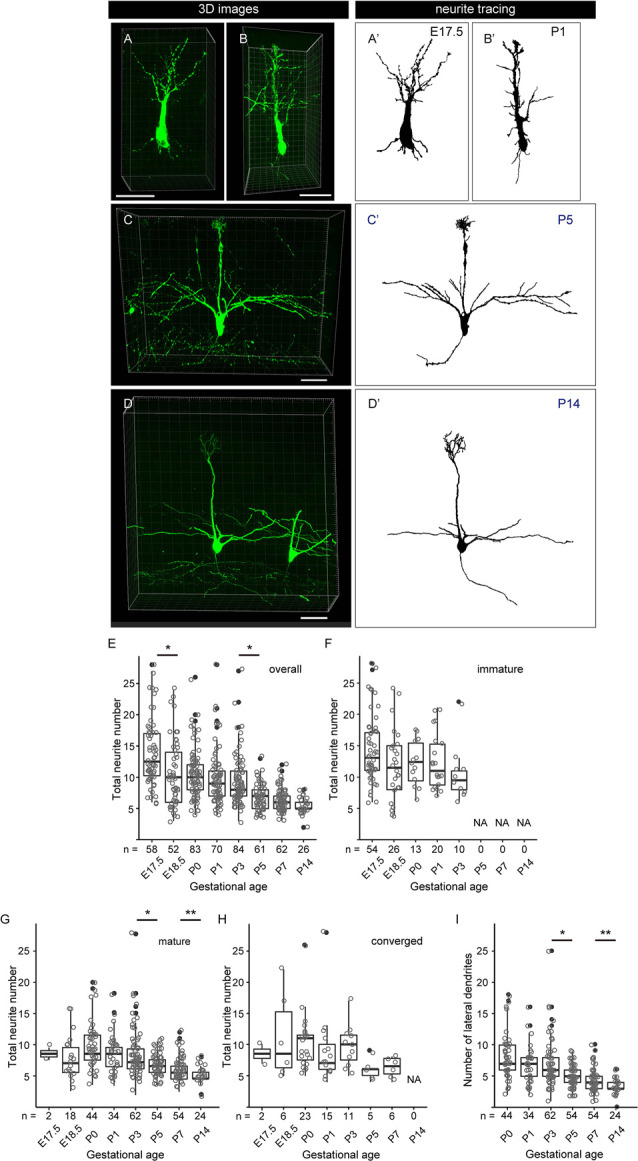
Quantitative analysis of neurite dynamics in developing mitral cells. **(A–D)** Representative snapshots of three-dimensional reconstructed confocal images of mitral cells and the corresponding traces **(A′–D′)** from each age are shown. Scale bars, 50 μm. **(E)** The age-dependent transition of neurite numbers in single mitral cells. The number represents the number of neurites arising from the soma, including both dendrites and axons, of single mitral cells. Statistical analysis was performed with the Kruskal–Wallis test followed by the Steel-Dwass *post hoc* test for multiple comparisons. Asterisks indicate significant differences (*P < 0.001). **(F–H)** The age-dependent transition of neurite numbers immature type **(F)**, mature type **(G)**, and converged type **(H)** mitral cells, respectively. Statistical analysis was performed with ANOVA + pair-wise Student’s *t-test* followed by FDR correction. The significance was accessed between adjacent ages. **(I)** Changes in age-dependent lateral dendrite numbers. Statistical analysis was performed with the Student’s *t-test* followed by Benjamini-Hochberg correction. Asterisks indicate significant differences (*P < 0.01, **P < 0.05). The white dots represent the entire data points, while the black dots represent outliers (outside the range of boxplot whiskers) that automatically come with the boxplots. The numbers of mice analyzed are 10, 4, 12 (five males and seven females), 14 (seven males and seven females), 10 (four males and six females), 7 (three males and four females), 7 (four males and three females), and 5 (two males and three females) for E17. 5, E18.5, P0, P1, P3, P5, P7, and P14, respectively. The sex of pups analyzed at E17.5 and E18.5 was not determined.

## Results

### Efficient AAV Transduction to Mitral Cells in Developing Mouse OB

To establish a novel method to visualize mitral cells in the mammalian olfactory system, we first examined whether the recombinant AAV could efficiently introduce the gene encoding EGFP into mitral cells in the mouse OB. The structure of the OB becomes apparent at ~ embryonic day 14.5 (E14.5) in the mouse developing the olfactory system, and we, therefore, reasoned that a single AAV injection directly into the olfactory ventricle near the OB at E14.5 could effectively target mitral cells. Indeed, when we injected AAV carrying EGFP into the olfactory ventricle at E14.5, we observed efficient and preferential labeling of mitral cells (Figure 1A). At postnatal day 14 (P14), we macroscopically checked the expression of EGFP over the whole brain and found that the fluorescence was predominantly observed within the OB (Figure 1B) and along with the LOT, which is formed by the axons of mitral cells (Figure 1C), suggesting that AAV preferentially transduces mitral cells. We next made coronal sections of the OB and observed them with a confocal microscope to assess the cell type specificity of AAV transduction. We found that most EGFP+ cells localized their somas within the mitral cell layer (MCL) and extended a single tufted neurite into the glomerulus layer (GL), which are all characteristics of mitral cells (Figures 1D–F). These observations suggest that, as expected from the macroscopic observation, AAV injection into the embryonic ventricle of the E14.5 mouse leads to predominant expression of EGFP in mitral cells (Figures 1D–F).

To further confirm the cell tropism of AAV, we performed immunohistochemistry using the antibody against Tbx21, a transcription factor that is exclusively expressed in the mitral/tufted cells in the mouse OB (Mizuguchi et al., 2012). In the OB at 3 weeks after birth, most EGFP^+^ cells (72.8%, *n* = 301) were localized in MCL, and 80.4% of them were Tbx21^+^ (Figures 1G–J), suggesting that the majority of EGFP^+^ cells are mitral cells. We also found EGFP^+^ and Tbx21^+^ cells in the external plexiform layer (21.3%, *n* = 301), which are probably tufted cells generated shortly after mitral cells with some temporal overlap (Batista-Brito et al., 2008). Also, 6.0% of EGFP^+^ and Tbx21^−^ cells were located in the inner layer, corresponding to granule cells. These data indicate that the majority of EGFP^+^ cells are mitral/tufted cells, and we thus conclude that AAV preferentially transduces mitral/tufted cells in the developing mouse OB.

We next tested multiple AAV serotypes including AAV-DJ, AAV2, AAV-DJ/8, and AAV-rh10, and found that all AAV serotypes we tested could efficiently transduce mitral cells (Figure 2). We utilized AAV-DJ for the later studies as we were able to prepare AAV-DJ particles with the highest yield.

### AAV Transduces Mitral Cells Independently of Birthdates

In the mouse OB, mitral cells develop through multiple rounds of cell division in an asynchronous fashion (Imamura and Greer, 2013). Therefore, labeling methods that can be applied to only a narrow window of cell division results in the visualizing of only a subset of mitral cells (Imamura and Greer, 2015). By contrast, given that AAV can transduce post-mitotic neurons (Haery et al., 2019), AAV-mediated gene transfer might label mitral cells in a birthdate-independent manner. To test this possibility, we injected 5-ethynyl-2′-deoxyuridine (EdU) intraperitoneally into pregnant mice carrying embryos at E9.5, E10.5, E11.5, E12.5, or E13.5 to analyze the relationship between EGFP-expressing mitral cells and their birthdates (Figure 3A). We then performed immunohistochemistry against the coronal sections of the P0 OB from each pup with antibodies against EGFP and Tbx21, followed by EdU detection. Consistent with previous reports, we found EdU signals in Tbx21^+^ cells in all sections that we observed regardless of the timing of EdU injection (Figures 3B–U). To further analyze the relationship between EGFP^+^ mitral cells (EGFP^+^/Tbx21^+^) and EdU signals, we calculated the percentage of EdU^+^ cells among EGFP^+^/Tbx21^+^ cells and found that the percentages were 11.7% (*n* = 145), 18.5% (*n* = 119), 33.6% (*n* = 113), 11.8% (*n* = 127), and 1.3% (*n* = 320) in mice in which EdU was applied at E9.5, E10.5, E11.5, E12.5, and E13.5, respectively (Figure 3V, Supplementary Figure 1). These data indicate that a single AAV injection at E14.5 can transduce mitral cells generated during E9.5–E13.5. Furthermore, the histogram pattern with a peak at E11.5 was proportional to that of the mitral cell genesis (Figure 3W). These findings demonstrate that, unlike *in utero* electroporation (Imamura and Greer, 2015; Muroyama et al., 2016), the AAV-mediated gene transfer method labels mitral cells independently of birthdates.

### Single-Cell Visualization of Mitral Cells

The specificity and birthdate-independence of the AAV-mediated gene transfer method further motivated us to attempt to visualize mitral cells in a single cell resolution. To this end, we applied the supernova system (Mizuno et al., 2014) to the AAV-mediated gene transfer method with several modifications (Figure 4A). In the original report, the supernova system was introduced by *in utero* electroporation of two plasmid vectors: one containing cDNA of the Cre recombinase under the tetracycline response element (TRE) and another containing a loxP-stop-loxP sequence with a bicistronic expression cassette of cDNAs for a fluorescent protein and tetracycline transactivator (tTA) combined with an IRES sequence under the CAG promoter (Mizuno et al., 2014). Because AAV has a limitation in the packaging size, we divided the bicistronic plasmid into two plasmids and used a double inverted open reading frame (DIO) cassette instead of the loxP-stop-loxP sequence for Cre dependent expression (Figure 4A). Also, we used the self-complementary AAV (scAAV) for the expression of Cre recombinase to accelerate the transduction speed (McCarty et al., 2001). Using this modified system, we successfully labeled a small number of mitral cells with EGFP in a single plane (Figure 4B). The high magnification image showed that EGFP fluorescence was bright enough to visualize not only the soma but also dendrites and axons (Figure 4C). These data indicate that AAV-mediated single-cell labeling provides a convenient system for visualizing the morphological details of individual mitral cells in the OB.

### Dendrite Remodeling of Mitral Cells in the Perinatal Stage

Using the AAV-mediated single-cell labeling technique, we next investigated the dendritic morphology of mouse mitral cells at P0. Mitral cells are thought to initially extend multiple dendritic branches to the glomerular layer and then eliminate all but one branch that innervates a single glomerulus (Lin et al., 2000; Blanchart et al., 2006; Sakano, 2020). According to this model, developing mitral cells are typically categorized into three subtypes based upon their dendrite morphology. Consistent with this model, we found mitral cells with three different morphologies; cells with multiple dendritic branches extending to the glomerular layer (Figure 5A; hereafter designated as “immature type”), cells with two main dendrites that innervate different glomeruli (Figure 5D; hereafter designated as “separated type”), and cells possessing a single primary dendrite whose terminals ramified within a single glomerulus (Figure 5C; hereafter designated as “mature type”). However, in addition to these conventional subtypes, we found mitral cells that have multiple primary dendrites emanating from a single soma, which converged onto a single glomerulus (Figure 5E; hereafter designated as “converged type”). This novel subtype of mitral cells with atypical dendrite morphology composed ~20% of mitral cells in the P0 mice OB (18.3%, *n* = 93).

Next, to investigate the dynamics of the dendritic morphology of mitral cells along perinatal development, we obtained images from cryosections of the OB at E17.5 (*n* = 83), E18.5 (*n* = 61), P0 (*n* = 93), P1 (*n* = 82), P3 (*n* = 109), P5 (*n* = 96), P7 (*n* = 72), and P14 (*n* = 46), respectively. Consistent with a previous report (Imamura et al., 2011), 95.2% of mitral cells at E17.5 extended multiple dendrites radially, corresponding to the immature type described above (Figure 5A). At E18.5, however, the percentage of the immature type mitral cells decreased to 50.8%, and accordingly, the mature type with a thick and tufted single primary dendrite emerged as 36.1% of the total population (Figures 5B,C). Subsequently, the percentage of the mature type of mitral cells continually increased, reaching ~90% of the total population at P7, whereas the immature type population continually decreased and eventually disappeared by P5 (Figure 5C), consistent with the conventional model (Lin et al., 2000; Wong and Ghosh, 2002; Blanchart et al., 2006).

The percentage of the separated type emerged as 3.3% of the total mitral cells at E18.5, and the percentage was gradually increased to ~15% of the total mitral cells at P14 (Figure 5F). By contrast, the converged type emerged as 9.8% of the total mitral cells at E18.5 (Figure 5F) and transiently increased to ~20% at P0, and then gradually decreased to 2.2% by P14. The dynamic changes in the converged type population with a peak at P0 suggest that the converged type might represent an intermediate stage in the transition from the immature type to the mature type.

Finally, we investigated the spatial relationship of each subtype and the location in the OB. To this end, we calculated the azimuth distribution from the actual mitral cell location in each coronal section from E17.5 to P3 mice and made scatter plots against mitral cells with four different dendrite types (Figures 5G–J). We then performed the statistical assessment using the Rao’s spacing test of uniformity (Rao, 1972, 1976) and found no significant deflection in the distribution of each mitral cell subtype in the OB (*n* = 153, *p* = 142.9; *n* = 169, *p* = 154.6, *n* = 53, *p* = 108.1, and *n* = 13, *p* = 134.3 for the mature type, the immature type, the separated type, and the converged type, respectively). These data suggest that the four subtypes of mitral cells categorized with dendrite morphology are evenly distributed in the mouse OB.

### Dynamic Changes of Neurite Numbers of Mitral Cells in the Perinatal Development

To further characterize the dendrite remodeling in mitral cells, we performed a quantitative analysis of developmental changes in the total number of neurites including axons and dendrites in single mitral cells. To this end, we quantified the numbers of neurites arising from the cell body in each 3D reconstructed image (Supplementary Figure 2). The representative 3D images and the corresponding traces in each developmental stage are shown in Figures 6A–D,A′–D′, respectively. Consistent with previous reports (Lin et al., 2000; Muroyama et al., 2016) and our population studies, most mitral cells established a thick and tufted single dendrite by P5 (Figure 5C). In the quantitative data of neurites, consistent with the data from the population changes of mitral cells (Figure 5), the average number of neurites from a mitral cell was gradually decreased from E17.5 to P14 and eventually reached ~3 (3.3 ± 0.5, *n* = 26) branches from a mitral cell (Figures 6E–I). These quantitative data further highlighted a reduction of the branch numbers in a mitral cell from E17.5 to E18.5, supporting the idea that the dendrite remodeling in mitral cells is triggered at least in part during the embryonic stage. Also, even after completion of the apical dendrite remodeling by P5, continuous reduction of the neurite number was observed until P14 (Figure 6E). Since a similar tendency was observed in the quantification of the lateral dendrites in the postnatal development (Figure 6I), likely, the reduction of the neurite numbers in the postnatal mitral cell development is at least in part due to the reduction of lateral dendrites. These observations suggest that dendrite remodeling in the lateral dendrites occurs with later developmental timing and/or takes longer time compared to apical dendrites.

## Discussion

In this study, we developed an AAV-mediated labeling system for mouse mitral cells in a single cell resolution. We first demonstrated that AAV injection into the olfactory ventricle at E14.5 leads to preferential gene transduction to mitral cells (Figure 1). This AAV-medicated cell labeling requires a relatively short period for gene expression as we found EGFP expression in mitral cells within 3 days by a single injection of AAV vector into the olfactory ventricle of E14.5 mouse. We examined multiple different AAV strains including AAV2, AAV-DJ, AAV-DJ/8, and AAV-rh10, and found no obvious difference in specificity as well as the efficiency of gene transduction to mitral cells (Figure 2). Because we used the ubiquitous CAG promoter that is strongly active in a wide range of cell types, the preferential transduction might be due to the tropism of the AAV vectors as AAV vectors show subtype-specific tropism even in a small brain region (Nathanson et al., 2009). It remains to be elucidated how AAV injection into the olfactory ventricle at E14.5 leads to preferential labeling of mitral cells. The early-born mitral cells are supposed to settle in the presumptive mitral cell layer of the OB at E14.5 (Imamura and Greer, 2013, 2015), and thus the axons, basal dendrites, and somata of the early-born mitral cells are proximal to the ventricle at E14.5, which makes them amenable to AAVs. It is also possible that AAVs would transduce progenitor cells in the proliferative zone surrounding the ventricle, which could contribute to the EGFP-positive mitral cells born after E14.5 (Imamura and Greer, 2015; Muroyama et al., 2016).

In addition to the efficient and specific gene expression, an important feature of the AAV-mediated gene transfer is the cell cycle-independent gene transduction (Haery et al., 2019). Indeed, the birthdate labeling using EdU revealed that AAV injection at E14.5 can transduce all mitral cell populations including the cells generated earlier than AAV injection (E9–E13; Figures 3V,W). Consistently, the spatial analysis indicated that the AAV-medicated gene transduction is unrelated to the localization of mitral cells in the OB (Figures 5G–J). This is in contrast to *in utero* electroporation-mediated cell labeling as *in utero* electroporation labels mitral cells generated after the timing of the gene electroporation (Imamura and Greer, 2015). Finally, by taking advantage of the highly efficient and preferential gene transduction in mitral cells as well as the independence of birthdates and localizations of mitral cells in OB, we established a single mitral cell-labeling system by combining the AAV technology with the Cre-mediated gene expression system (Figure 4).

**Figure 7:**
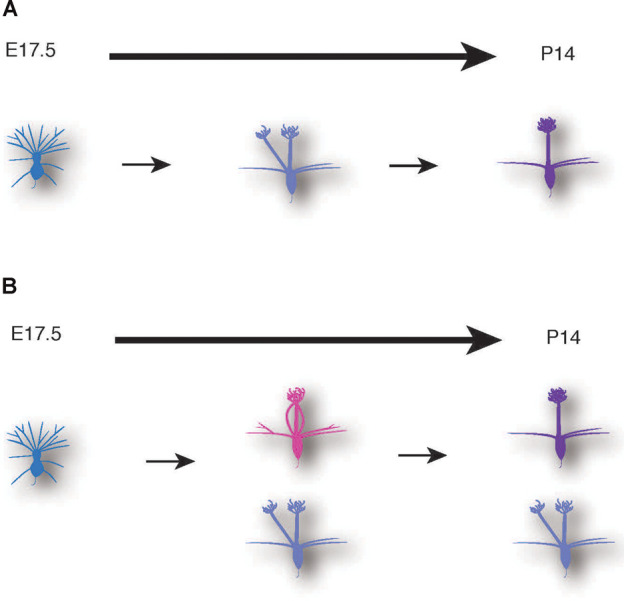
Schematic illustrations of mitral cell development. A conventional model **(A)** and a novel model based on our data **(B)** of dendrite remodeling in the developing mouse mitral cells.

The AAV-mediated single-cell labeling allowed us to analyze the dynamic aspects of developing mitral cells quantitatively. As the first example, we applied this AAV technology to detailed studies of dendrite remodeling in mitral cells in the perinatal development. Using AAV technology, we found two novel points concerning the dynamics of dendrite remodeling. First, we found a novel subtype of mitral cells that extended multiple dendritic branches to the same glomerulus (Figure 5). This atypical population accounted for ~20% of mitral cells in the OB at P0 (Figure 5). To our knowledge, such a converged innervation of mitral cell dendrites in a single glomerulus has not been described in the previous reports. Indeed, the population of the converged type was gradually decreased and eventually disappeared by P14 at the time when dendrite remodeling is completed (Figure 5F). It is thus likely that the converged type might represent a transient form of dendrite remodeling from the immature type to the mature type (Figure 7). We could not rule out the possibility that tufted cells might contribute to the population changes of EGFP-labeled cells (Figures 5C,F), as AAV injection at E14.5 likely labels tufted cells as we as mitral cells (Figure 1). Also, our quantitative analysis revealed that over 10% of mitral cells have separated-type dendrites in P14 mice (Figure 5F). In the non-mammalian vertebrate OB such as amphibians and reptiles, mitral cells extend apical dendrites to multiple glomeruli (Imamura et al., 2020), but to our knowledge, no such multi-innervating mitral cells were described in the mature mammalian OB. The role of mitral cells with the separated-type dendrites in mature animals is currently unknown. Considering that the separated-type cells likely gain neural information from multiple glomeruli, they might contribute to odor information processing including information integration in the olfactory circuits. Future genetic and electrophysiological studies on the separated type mitral cells will help to understand their roles in the olfactory circuits.

The other novel finding is the developmental time frame of dendrite remodeling in mitral cells. We found that ~50% of mitral cells completed their dendrite remodeling in the late embryonic stages (E17.5–E18.5). Considering that the dendrite remodeling in the embryonic stage has not been reported in previous studies, the population might be hardly labeled by conventional methods such as *in utero* electroporation and the retrograde labeling by lipophilic dyes delivered *via* the LOT. The *in utero* electroporation for mitral cell labeling is typically performed at E10–12 (Imamura and Greer, 2015; Muroyama et al., 2016), which is thus unlikely to label mitral cells generated in E9–E10. By contrast, our quantitative data suggest that over 60% of the AAV-mediated EGFP positive mitral cells are born from E9 to E11 (Figures 3V,W). As for the retrograde labeling by lipophilic dyes delivered *via* LOT, a recent article showed that the axonal path of the early-born and the late-born mitral cells are segregated in a sublamina manner: axons of the early-born mitral cells are localized in the deep sublamina, whereas the axonal path of the late-born mitral cells is restricted in the most superficial sublamina of LOT (Imamura and Greer, 2015). Given that the superficial axonal layers are supposed to be closer to lipophilic dyes compared to the deeper axonal layers, the late-born mitral cells might be preferentially labeled by the retrograde labeling with lipophilic dyes delivered through LOT. It is thus possible that the mitral cell populations that obtain a matured dendritic branch before the birth account for the populations generated in the earlier embryonic stages. Consistent with this notion, in the birthdate labeling studies (Figure 3), 87.5% (14 of 16) of mitral cells with birthdate labeling at E9.5 had a mature apical dendrite at P0, whereas 58.3% (7 of 12) of mitral cells with birthdate labeling at E11.5 had a mature apical dendrite at P0.

Our findings also suggest that the dendrite remodeling in mitral cells is triggered by mechanisms independent of the odor-evoked activity in mitral cells because the odor-evoked activity is typically observed after birth in the mammalian sensory circuits (Brennan et al., 1990). This notion is consistent with the previous reports that dendrite remodeling in mitral cells was largely unaffected in mice lacking function of the olfactory cyclic nucleotide-gated (CNG) channels that are required to evoke odor-triggered signaling in mitral cells (Lin et al., 2000) although a small delay in the early postnatal stage (P4–P6) was observed. A similar dendrite remodeling delay was observed in mice lacking Sema7A and its potential receptor PlexinC1 (Inoue et al., 2018). Given that Sema7A is likely to be induced by the odor-evoked activity in olfactory sensory neurons and that Sema7A and PlexinC1 function in synapse formation (Inoue et al., 2018), odor-evoked activity in mitral cells might contribute to dendrite remodeling in part through synapse formation and/or stabilization between sensory neuron axons and mitral cells dendrites. Therefore, our data, together with the previous reports, support the idea that the odor-evoked activity in mitral cells is dispensable and rather play a permissive role in the dendrite remodeling.

What mechanisms could drive dendrite remodeling in mitral cells in the embryonic stages? Given that dendrite remodeling in the early-born mitral cells likely proceeds in the earlier timing compared to the late-born mitral cells (Figure 4C), the timing of dendrite remodeling might be triggered in part by a genetic program that mitral cells obtain when they are born. Indeed, in the Drosophila olfactory system, wiring between the olfactory sensory axons and dendrites of the second-order projection neurons is established mostly by genetic control through multiple transcriptional factors (Hong and Luo, 2014). Besides, dendrite remodeling in Drosophila sensory neurons during metamorphosis is also independent of neural activity. Instead, dendrite pruning is triggered by the steroid hormone Ecdysone and its cognate receptor, which in turn transcriptionally induces expression of multiple components required for dendrite pruning, such as ion channels, ubiquitin proteosome-related components, and cytoskeletal regulators (Kanamori et al., 2013; Yu and Schuldiner, 2014; Furusawa and Emoto, 2020). It is thus possible that the dendrite remodeling in mitral cells might share a part of the transcriptional program as well as the transcriptional targets with those in Drosophila sensory neurons.

An alternative scenario is that spontaneous activity in the olfactory sensory system might contribute to dendrite remodeling. In many sensory systems, a spontaneous activity often emerges before the timing when sensory-evoked activity is observed, and shape the wiring of emerging circuits (Blankenship and Feller, 2010; Kerschensteiner, 2014). For example, retinal waves in the mammalian visual cortex are required for functional refinement of visual circuits (Ackman et al., 2012). Similarly, in the mouse somatosensory system, patchwork-type spontaneous activity is observed in layer 4 neurons in the postnatal somatosensory cortex although its function is unknown (Mizuno et al., 2018). In the Drosophila olfactory system, spontaneous activity is likely required for proper sensory processing and behavior (Utashiro et al., 2018). A recent report suggests a potential role of spontaneous activity in dendrite remodeling in mitral cells (Fujimoto et al., 2019). Further studies will be required to examine the role of spontaneous activity in the development and function of the mammalian olfactory system. It might be worth noting that the compartmentalized calcium (Ca^2+^) transients in dendritic branches trigger dendrite pruning in Drosophila sensory neurons (Kanamori et al., 2013, 2015). It is thus of interest to monitor Ca^2+^ dynamics in dendritic branches in the developing mitral cells.

In summary, we have described a novel AAV-mediated labeling system for mitral cells in a single cell resolution. This AAV technology complements the current labeling techniques such as dye injections and *in utero* electroporation and contributes to a better understanding of functional organizations of the mouse olfactory circuits. Given the high efficiency and preference in gene transduction to mitral cells, in addition to overexpression or suppressing of a gene of interest in mitral cells, the AAV-mediated gene transduction can be applied to the manipulation of mitral cell activity in the functional olfactory circuits. This will provide an excellent platform to address dynamic aspects in developing mitral cells as well as functional aspects in matured mitral cells.

## Data Availability Statement

The raw data supporting the conclusions of this article will be made available by the authors, without undue reservation.

## Ethics Statement

All animal experiments were carried out in accordance with the regulations and guidelines for the care and use of experimental animals at the University of Tokyo and were approved by the University of Tokyo Graduate School of Science.

## Author Contributions

KT, MT, and KE: conceptualization. KT and MT: methodology and formal analysis. MT: software. KT, ST, and RN: validation. KT and KE: resources. HK, ST, RN, and MT: data curation. KT: writing an original draft. KE: writing—review and editing, supervision, project administration, and funding acquisition. All authors contributed to the article and approved the submitted version.

## Conflict of Interest

The authors declare that the research was conducted in the absence of any commercial or financial relationships that could be construed as a potential conflict of interest.

## Footnotes

^1^http://n2t.net/addgene:37825

^2^http://n2t.net/addgene:18783

^3^http://n2t.net/addgene:51904

^4^http://n2t.net/addgene:32396

## Supplementary Material

SUPPLEMENTARY FIGURE 1 The ratio of EdU-labeled cells in EGFP- and Tbx 21- positive cells. **(A)** The timing of administration of EdU and the ratio of EdU-labeled cells in EGFP-positive and Tbx 21 positive cells. The numbers of mice analyzed are 1, 1, 3, 1, and 3 for E9.5, E10.5, E11.5, E12.5, and E13.5, respectively. **(B)** The timing of administration of EdU and the ratio of EdU-labeled cells in Tbx21-positive cells. The numbers of mice analyzed are 1, 2, 2, 2, and 2 for E9.5, E10.5, E11.5, E12.5, and E13.5, respectively. The average percentage (blue bars) and the distribution of mice analyzed (circles) are shown.

SUPPLEMENTARY FIGURE 2 Three-dimensional reconstructed confocal images of mitral cells. Images **(A–D)** corresponds to Figure 6 **(A–D)**. Images **(A,A′)** and images **(B,B′,B′′)** are views from different angles of the same neurons. Blue and white arrowheads represent axons and lateral dendrites, respectively.
