# Supplementary figures and images for: Adeno-Associated Virus-Mediated Single-Cell Labeling of Mitral Cells in the Mouse Olfactory Bulb: Insights into the Developmental Dynamics of Dendrite Remodeling
Source: Front Cell Neurosci. 2020 Dec 9;14:572256. doi: 10.3389/fncel.2020.572256 (PMC7756102; doi:10.3389/fncel.2020.572256)

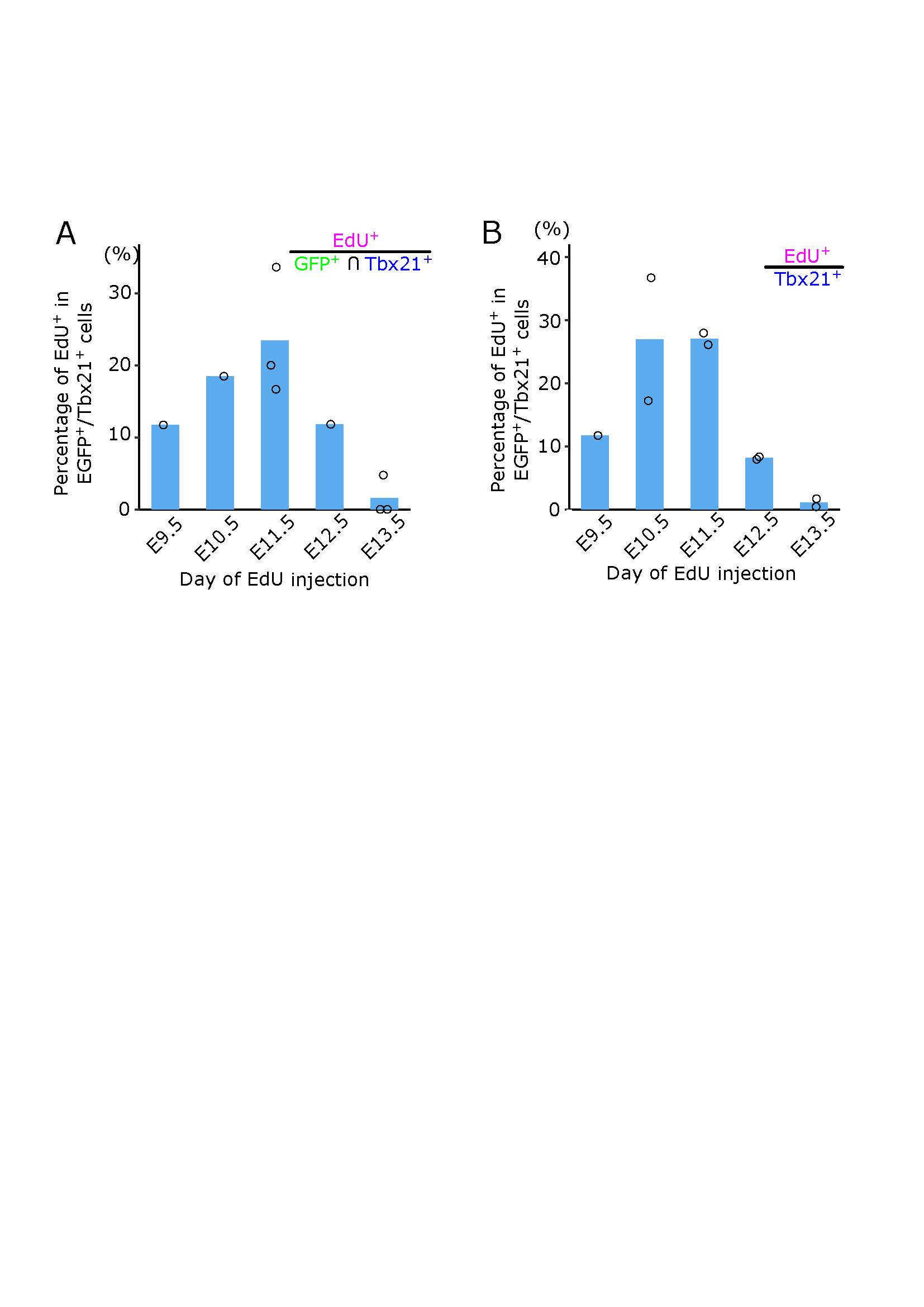

Supplement: SUPPLEMENTARY FIGURE 1 — The ratio of EdU-labeled cells in EGFP- and Tbx 21- positive cells. (A) The timing of administration of EdU and the ratio of EdU-labeled cells in EGFP-positive and Tbx 21 positive cells. The numbers of mice analyzed are 1, 1, 3, 1, and 3 for E9.5, E10.5, E11.5, E12.5, and E13.5, respectively. (B) The timing of administration of EdU and the ratio of EdU-labeled cells in Tbx21-positive cells. The numbers of mice analyzed are 1, 2, 2, 2, and 2 for E9.5, E10.5, E11.5, E12.5, and E13.5, respectively. The average percentage (blue bars) and the distribution of mice analyzed (circles) are shown. [file Image_1.JPEG]

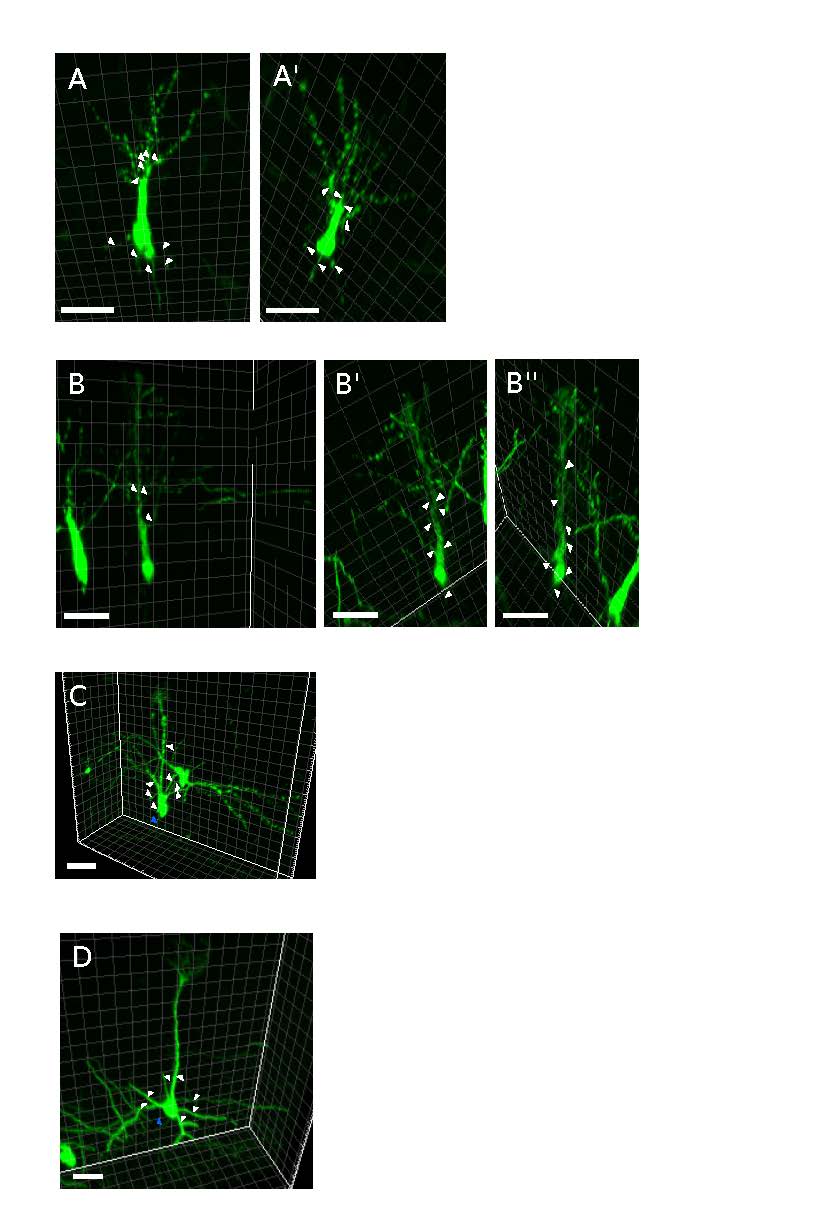

Supplement: SUPPLEMENTARY FIGURE 2 — Three-dimensional reconstructed confocal images of mitral cells. Images (A–D) corresponds to Figure 6 (A–D). Images (A,A′) and images (B,B′,B′′) are views from different angles of the same neurons. Blue and white arrowheads represent axons and lateral dendrites, respectively. [file Image_2.JPEG]
